# Chronic Depression Alters Mothers’ DHEA and DEHA-to-Cortisol Ratio: Implications for Maternal Behavior and Child Outcomes

**DOI:** 10.3389/fpsyt.2020.00728

**Published:** 2020-07-22

**Authors:** Yael Apter-Levy, Orna Zagoory-Sharon, Ruth Feldman

**Affiliations:** The Center for Developmental Social Neuroscience, Interdisciplinary Center Herzliya, Herzliya, Israel

**Keywords:** HPA, maternal depression, cortisol, dehydroepiandrosterone, longitudinal studies

## Abstract

Maternal depression is a major public health problem that typically occurs in the period surrounding childbirth. The neurobiological mechanisms underlying maternal depression have been the focus of increasing research and studies pointed to the crucial role of the HPA axis in this disorder. However, most studies focused on cortisol expression and regulation while recent attention has shifted to include the sulfate steroids DHEA and DHEA-S. A community cohort of 1,983 women with no comorbid risk was recruited at birth and depression was assessed periodically across the first postpartum year. At 6 years, 156 families were re-visited: 46 mothers were defined as chronically-depressed and 103 controls reported no depression from birth to six years. Mothers and children were diagnosed by structured psychiatric interviews and mother-child interactions were observed. Maternal diurnal cortisol (CT) and dehydroepiandrosterone (DHEA) were assessed. Depressed mothers had lower levels of DHEA (AUCg), flattened DHEA diurnal variability (AUCi), and smaller DHEA-to-CT Ratio. Regression analysis demonstrated that maternal sensitivity during mother-child interaction was independently predicted by maternal depression, DHEA levels, child CT, and child social withdrawal. Results underscore the need for multi-level understanding of the dynamic interplay between maternal psychopathology, mother-child relationship, and pituitary–adrenal-cortex-to-medulla balance in studying the cross generational transfer of psychiatric vulnerability from depressed mothers to their children.

## Introduction

Maternal Depression is a common condition and constitutes a major public health problem. During pregnancy and the postpartum period the body is more vulnerable to many different disorders including hypertension, diabetes, and cardiovascular disease, in addition to high stress and psychopathology (1). One of the most investigated mechanisms of depression is dysfunction of the HPA Axis. The functioning of the HPA system is thought to be shaped in prenatal and early neonatal life through a variety of proximate conditions, including maternal stress physiology, contextual conditions, and parenting quality (2–4). Additionally, the HPA axis is thought to play a crucial role in the initiation and maintenance of depressive illness. The most thoroughly investigated aspect of the HPA axis are variations in the secretion of the stress hormone Cortisol (CT). During the days and weeks after birth, there is a fall in cortisol and CRH (Cortisol Releasing Hormone), which is especially marked in women suffering from postpartum depression (5, 6). Glynn et al. proposed that depressive postpartum symptoms may be due to prenatal HPA axis dysregulation (7, 8).

However, CT is not the only stress-related hormone associated with depression and malfunctioning and its potential impact on offspring psychopathology. The adrenal androgen dehydroepiandrosterone (DHEA) plays a critical role in controlling mood and anxiety, and changes in DHEA levels have been reported in conditions pertaining to increased stress and psychiatric disorders (9, 10). It had been recognized as early as 1952, that lower DHEA/DHEAS in adult life is associated with neuropsychiatric disorders (eg schizophrenia, depression). However, the mechanistic role for DHEA/DHEAS in any of these domains remains speculative, not the least because the presence of these androgens in the adrenal gland and brain is largely confined to humans and a few non-human primates. DHEA and DHEAS are dynamically regulated from before birth and before the onset of puberty, and therefore an understanding of the synthesis, regulation, and functions of this important androgen pathway warrants attention. Davies (11) has stressed the important role of the steroid sulfate axis for maternal mental health and there evidence suggesting that maternal caregiving may affect the psychological adjustment of offspring *via* these hormonal mechanisms. Furthermore, these hormones seem to have some therapeutic value (11). In a recent meta-analysis, there seem to be a significant treatment effect for these hormones in depression when compared to placebo (12) and DHEA and DHEA-s were found to influence basic electrophysiological processes. An interest finding in this regard is that depression is accompanied by an attenuated DHEA and DHEA-S response to acute psychosocial stress, which may help elucidate the relationship between the HPA axis and stress (13). Thus, higher DHEA-to-cortisol and DHEAS-to-DHEA ratios are hypothesized to be involved in negative stimuli processing, preventing the interference of negative stimuli in cognitive tasks. DHEA-S may play important roles in cortical development and plasticity, protection against negative affect and depression, and might even enhance attention and overall working memory (14). Alterations in DHEA activity seem to be trait rather than state phenomena and are predictive of future depressive episodes (15–17). Additionally, there is evidence of anatomical structural changes in the brain related to these findings. Thus, both pituitary (18) and hippocampal volume appears to be reduced in depressive patients (16).

Maternal depression, both antenatal and postnatal, is associated with reduced maternal sensitivity to offspring (19). In a recent prospective study, it was found that the presence of depressive symptoms augmented deficits in maternal sensitivity in mothers who suffer from personality disorders (20). Moreover, poor quality mother-infant interactions in the perinatal period predicted is associated with suicidal ideation in pregnancy (21). In a recent meta-analysis, the association of reduced maternal sensitivity to maternal depression was clearly demonstrated, and it may be that the pernicious influence of maternal depression on child development is mediated by maternal sensitivity (22). The HPA Axis appears to play a seminal role in maternal sensitivity (7). For example, results from the Alberta Pregnancy Outcomes and Nutrition Study, (a prospective longitudinal cohort of pregnancy), suggested that maternal HPA axis is a means by which early life stress in mothers is transmitted to their children. The authors point out that their results show that the HPA axis is sensitive to social stimuli (23). However, although much research has been devoted to study mechanisms by which cortisol changes are associated with depression (1), very little research has been devoted DHEA and no study, to our knowledge, has focused on DHEA in the context of maternal depression and the mother’s observed caregiving.

Thus, the overall goal of this prospective longitudinal study was to examine the vicissitudes of DHEA/CT ratio in depressed mothers of preschoolers in a community cohort followed from birth to six years. Two hypotheses were proposed; First, depressed mothers will have lower DHEA levels than mothers without depression in combination with a less dynamic DHEA system (24), and that DHEA-to-CT ratio will be smaller for the depressed group (16). Second, interactions between depressed mothers and their children would be expected to be less optimal, marked by lower maternal sensitivity and greater child social withdrawal during social contact. Consistent with ecological models on the determinants of sensitive parenting (25), which suggested that both child biological factors and parental characteristics contribute to the development of sensitive parenting, in addition to recent evidence that child and maternal cortisol are inter-correlated in the context of maternal depression (26) we expected that maternal hormones and depression will predict the degree of maternal sensitivity during naturalistic interactions with her child.

## Methods

**Figure 1:**
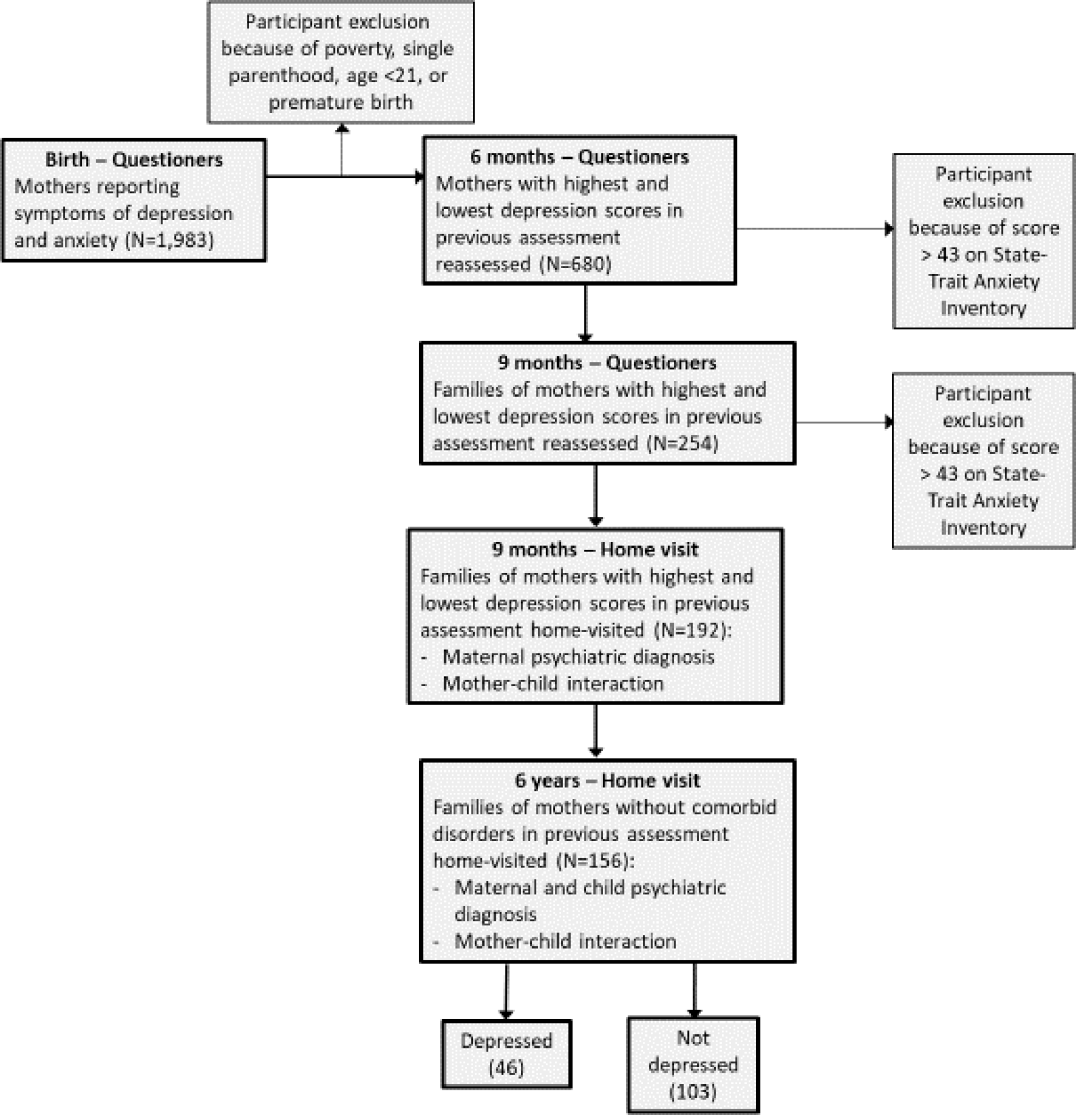
Flow chart depicting sequence of assessments.

Methods of the study have been reported extensively in our previous publications, and are summarized here briefly (27, 28). We included **Figure 1**, which details the five ways of sample recruitment and follow-up from birth to six years, including the exact number of participants at each wave and numbers lost to attrition.

### Participants

Participants were recruited in five waves of data collection utilizing an extreme-case design as follows: 1,983 consecutive admissions to maternity ward were included in the initial sample. Inclusion criteria were physically healthy mothers, a healthy, term, and singleton infant, and stable family situations with low-risk socio-economic status: mother age above 21 years, completed high-school education, cohabitating with infant father, and family above poverty line. The initial assessment included demographic questionnaires, BDI [Beck Depression Index (29)] and STAI [State-Trait Anxiety Inventory (STAI) (30)]. Six months later the mothers were reassessed and divided to those with high BDI scores (>11) and low scores (0–5), respectively. At nine months, we approached 350 mothers for reassessment, of which 254 responded. Of the 254 mothers who responded at nine months, 210 were contacted who had high and low depressive symptomatology without high anxiety symptoms (STAI>43). Of those, 192 agreed to a home visit, which included the Structured Clinical Interview for DSM-IV Axis I Disorders (SCID-I; (31). At all stages of the evaluation there were no differences in the demographic characteristics between those who agreed or disagreed to participate in the study. At 6 years, 156 mothers and children were revisited. At this stage, two final study groups of mothers remained: those with high depressive symptoms from birth (n=46), and those with no psychiatric diagnosis from 9 months to 6 years. No differences in demographic factors were found between these two groups (**Table 1**). The study was approved by the Institutional Review Board and all participants signed an informed consent.

**Table 1:** Demographics information for depressed and non-depressed groups.

	Non-depressed		Depressed		Statistics
	Mean	SD	Mean	SD	*T* value
**Mother education**	14.24	2.54	13.97	2.78	0.54, ns
**Mother age (years)**	37.05	4.12	35.74	4.9	1.76, ns
**Child age (months)**	76.51	24.69	71.33	13.46	1.84, ns
**Child gender**					*χ*^2^(5) = 0.25, ns
**Male%**	52.7%		57.5%		54.2%
**Child birth order**					*χ*^2^(1)=3.50, ns
**Firstborn%**	36.2%		54.1%		41.1%

### Procedure and Measures

All interviews were conducted at home, in the afternoon, after 3:00PM. Mothers were administered the DAWBA (32) and then the SCID (31), and the child was evaluated with a neuropsychological test, the NEPYS and Vocabulary part and Block sub-test from the WIPPSI. In addition, the children underwent emotion regulation and empathy paradigms. Mother and child were videotaped in a 10-min interaction with age-appropriate pre-selected toys. Mothers were given 12 tubes for diurnal salivary samples collection from themselves and the child (3 per day) by passive drool on two consecutive weekend days of the same week.

Maternal Psychiatric Diagnosis was by SCID-I (31), 46 mothers (29.6%) were defined as chronically depressed. These mothers showed high depressive symptoms (BDI >11) at birth, six, and nine months, received a clinical diagnosis of MDD at both nine months and 6 years, and reported being depressed throughout most of the child’s first six years. One hundred and three mothers (66%) non-symptomatic mothers formed the control group,

#### Hormone Collection and Analysis

##### Cortisol and DHEA Diurnal Collection

Participants were provided with detailed instructions for collecting saliva. Samples were collected upon wakening, 30 min after wakening, and before going to bed. Mothers and children were asked to chew on salivates (Sarstedt, Rommelsdorft, Germany) until saturation on two consecutive days. Mother received salivates for cortisol and DHEA assays, and the children for cortisol alone. Participants kept a collection diary in which they noted exact awakening and sampling times for each sampling day. Salivates were stored at −20°C until analysis. Child results are reported elsewhere (28).

*Cortisol Analysis* was done using standard procedures and described in our original paper (27).

*DHEA Analysis* was done using standard procedures described by our group elsewhere (33): In order to precipitate the mucus, samples underwent several freeze-thaw cycles. After the fourth cycle the tubes were centrifuged at 1,500 x g (@3,000 rpm) for 20 min. Supernatants were collected and stored at -20°C until assayed. Determination of DHEA was performed using a commercial DHEA ELISA kit (Salimetrics, USA). Salimetrics DHEA kit is a competitive immunoassay specifically designed for the quantitative measurement of salivary DHEA. On the day of assay, samples were thawed, and 50 micro-liters were pipette into the appropriate well of the kit. Measurements were performed in duplicate and the concentrations of samples were calculated by using MatLab-7 according to relevant standard curves. The intra-assay and inter-assay coefficients are 20.9 and 22.7 percent, respectively.

Two measures were calculated for diurnal CT and DEHA: area under the curve with respect to ground (AUCg) and area under the curve with respect to increase (AUCi) (34). The AUCg is an estimate of the total diurnal CT or DHEA secretion over six measurements (3 times a day, for two consecutive days: waking, noon and evening) (28). The AUCi is a measure of the dynamic increase of diurnal secretion, associated with the variability and sensitivity of the system and emphasizing changes over time during the days (34).

#### Mother-Child Interaction

10 min of mother-child interaction with a set of pre-selected toys were filmed and coded with the Coding Interactive Behavior (CIB) manual [for review: (35)], a coding system that has shown good psychometric properties and have been utilized across the world in research spanning infancy to adulthood. We have described this procedure in previous reports (36). In brief, interactions are coded offline on 52 scales that are combined into eight theoretically-determined maternal, child, and dyadic constructs. In the current study, we focused on the construct of maternal sensitivity, which includes codes related to the expression of maternal behavior (e.g, positive affect, warm vocalizations, continuous social gaze), adaptation to the child’s state and signals (e.g., appropriate range of affect, resourcefulness), and the provision of a secure base (e.g., maternal supportive presence). Maternal sensitivity is the key construct that describes the mother’s growth-promoting style in attachment research. The CIB maternal sensitivity construct has been shown to be individually stable from infancy to adolescence, associated with a host of positive child outcomes, and compromised in multiple high-risk conditions (35). In addition, we used the CIB *Child Social Withdrawal*, which comprises codes related to child avoidance, distancing from mother, negative/withdrawn mood, and minimal social involvement and has shown to be altered in children of depressed mothers (37, 38).

### Statistical Analysis

Differences between depressed and non-depressed mothers and their children in diurnal and reactive hormone levels and behavior were tested with ANOVA. Hierarchical multiple regression was used to predict maternal behavior by maternal depression and maternal and child hormonal profiles.

## Results

### Group Differences in Hormonal Profiles and Interactive Behavior

#### Hormones

**Figure 2:**
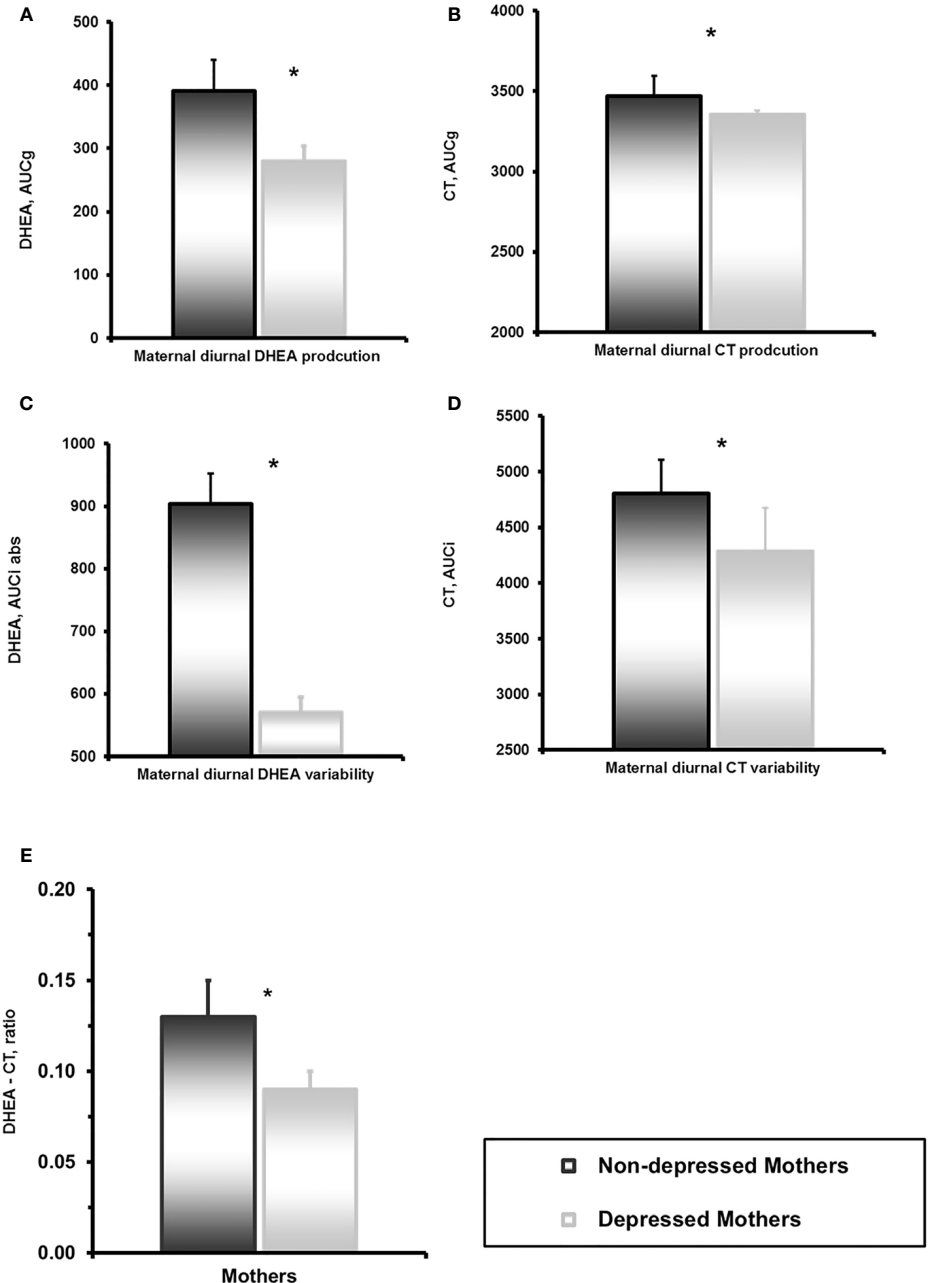
**(A)** Mother’s dehydroepiandrosterone (DHEA) diurnal secretion in depressed and non-depressed mothers at 6 years. *p<0.05. **(B)** Mother’s and child’s cortisol diurnal secretion in children of depressed and non-depressed mothers at 6 years. *p<0.05. **(C)** Mother’s DHEA diurnal variability in depressed and non-depressed mothers at 6 years. *p<0.05. **(D)** Mother’s and child’s cortisol diurnal variability in children of depressed and non-depressed mothers at 6 years. *p<0.05. **(E)** DHEA-CT ratio in depressed and non-depressed mothers at 6 years. *p<0.05.

Depressed mothers showed significantly lower levels of total diurnal DHEA secretion, lower variability of DHEA, and smaller DHEA to CT Ratio compared to controls (**Table 2**, **Figures 2A–D**). We have reported elsewhere on child CT levels. In short diurnal cortisol secretion in mothers was associated with that of the child (39).

**Table 2:** Differences in hormonal measures and maternal sensitivity according to maternal depression.

	Depressed Mothers		Non-Depressed Mothers		T
	Mean	SEM	Mean	SEM	
**Maternal DHEA AUCg**	279.35	24.38	391	48.29	2.07*
**Maternal DHEA AUCi**	-570.02	59	-903.89	187.25	1.701*
**Maternal DHEA-CT**	.03	.02	.09	.01	1.8*
**Child CT AUCg**	3777.85	227.95	2910.26	80.27	3.59**
**Child CT AUCi**	-3784.84	217.78	-3140.97	252.98	1.93*
**Maternal Sensitivity**	3.37	0.13	3.75	.06	2.65*
**Child Withdrawal**	1.5	.11	1.21	.03	-2.56*

Differences between depressed mothers and non-depressed mothers, and their children in means and SEM of mother’s and child’s diurnal cortisol and DHEA secretion and variability and maternal sensitivity and child withdrawal CIB scores at 6 years *p < .05 **p < .01.

#### Interactive Behavior

Depressed mothers showed less sensitivity during mother-child interaction compared to non-depressed mothers. Children of depressed mothers were more withdrawn during interactions with their mother compared to children of non-depressed mothers (**Table 2**).

Maternal Sensitivity was negatively associated with Child Withdrawal during mother-child interaction, r=-.381, p<.001. Maternal Sensitivity correlated with higher mother’s (AUCg) diurnal DHEA production, r= 0.157, p<.05 and diurnal DHEA variability (AUCi) r=.187, p<.05.

### Predicting Maternal Sensitivity From Maternal Depression and Mother and Child Hormones

A hierarchical multiple regression was computed to predict maternal sensitivity from maternal and child stress-related hormones in the context of chronic maternal depression. Variables were entered in four theoretically-determined order to test the unique effect of neuroendocrine markers in mother and child above and beyond the effect of depression. In the first, step, maternal depression was entered to control for its effect on maternal sensitivity. In the second block, maternal DHEA level (AUCg) and variability (AUCi) were entered, and in the third block, child CT level (AUCg) and variability (AUCi) were entered. In the final block, child social withdrawal during mother-child interaction was entered to address the impact of child social involvement and withdrawal on the mother’s sensitive behavior. Results regression model appear in **Table 3** and detail the four steps of the model. As seen, each step contributed meaningfully to the prediction of maternal sensitivity, indicating that maternal depression, maternal and child stress-related neuroendocrine markers, and child social behavior jointly impact the mother’s sensitive style. In combination, the variables included in the model explained 43% of the variability in maternal sensitivity.

**Table 3:** Hierarchal regression analysis predicting maternal sensitivity correlates.

		*B*	*SEB*	β	*Adjusted R^2^*
Step 1					
	Maternal Depression	-.64	.16	-.42^**^	.17
Step 2					
	Mother DHEA AUCg	-2.51	1.10	-.57^*^	
	Mother DHEA AUCi	.38	.29	.27	.23
Step 3					
	Child CT AUCg	-.48	.64	-.27^*^	
\	Child CT AUCi	.15	.09	.16	.28
Step 4					
	Child withdrawal	-.52	.15	-.35^**^	.38

R^2^ Total = .43 F_(6,65)_ = 8.443, p <. 001.

*p < .05 **p < .001, sample size=137 mother and child.

## Discussion

The major novel finding of this study was the close association between maternal depression and maternal DHEA hormonal activity. It may be that elevated stress activates the HPA axis either as a result of maternal depression or alternatively as a cause of maternal depression. Our results point to dysregulation of maternal HPA functioning as expressed by disturbances in DHEA secretion and the DHEA-to-Cortisol ratio. As expected, mothers with a chronic history of depression showed flattened diurnal curves. The most common explanation for such flattened curve is that an “allostatic overload” of the HPA system from exposure to prolonged stress leads to exhaustion of the system, resulting in inflexible hormonal production (40). Interestingly, similar findings have been shown with patients suffering from “Burnout” (41), and also from a history of physical abuse in early life (42).

The concept of “allostatic load” has received increasing attention and some researchers have even recommended placing it alongside in importance to traditionally recognized cardinal factors such as genes and the environment (43). Although in the past interest in “allostatic load” has mainly been limited to CT expression, it now seems that the allostatic load story “is a tale of two axes” (44). The second axis being the sulfate steroid axis, and our mothers with a history of depression showed both lower levels and flatter diurnal curves of DHEA. There is much evidence to show that the DHEA to CT ratio has a pivotal role in depression particularly in a developmental context, as has been recently reviewed (45). The authors concluded that absolute and relative hormone levels of DHEA and CT may be relevant in understanding developmental psychopathology and the two hormones may have opposing effects, and speculate that these hormones may be the basis for developing biomarkers that are relevant when making a clinical diagnosis. Interestingly, CT to DHEA ratio have been found to be markers of emotional resilience in rats in animal models, independent of the presence or absence of depression (46). This ratio was altered following reproductive experience. Thus, this endocrine ratio may be particularly relevant for maternal depression (47).

Our findings also support Girdler et al. (24) suggestion that neuroactive steroids are mainly synthesized in the adrenals and that a history of depression may be associated with persistent adrenal suppression (24). Indeed, we show that the DHEA to CT ratio was significantly lower in depressed mothers compared to controls; it seems that DHEA secretion was even more sensitive than CT to allostatic pressures in depressed mothers, a finding that may be of interest when developing new therapeutic strategies for chronic maternal depression (9, 12). There is some evidence that the beneficial effects of these agents are mediated by the regulation of the HPA axis (48). Depressed mothers tend to respond less sensitively to their infants’ signals (49), which in turn may affect the infants’ ability to regulate stress and negative emotions (38) and engage in social interactions (50). Results of the regression model from the current study may shed further light on this interplay among the different levels of functioning. Maternal sensitivity during interaction with her child was predicted by the mother’s depression, her DHEA levels, and her child’s withdrawal behavior and cortisol levels. Taken in conjunction with the association between low child cortisol secretion and variability and child withdrawal when interacting with the mother, we can surmise that the interplay between cross- generational HPA function, mother-child relationship, child behavior, and maternal depression are all inextricably intertwined leading to greater child psychiatric vulnerability (51, 52). A recent study, (3) showed an association between maternal depression and infant HPA axis sensitization. In this study, CT reactivity was increased and also magnified over time. This pattern of response predicted maternal depressive disorder, which in turn was related to poorer infant development. It has been reported that mothers’ sensitivity is related to bonding and social and emotional behavioral problems (53). Thus, maternal factors impacting the quality of mother-child interaction are important for children’s positive social-emotional development.

It is still uncertain as to whether hyperactive stress responsivity is associated with affective disorder *via* changes occurring overtime due to chronic stress response and whether these findings will fit a classic “allostatic load” model which indicates that elevated HPA activity results in accumulated “wear and tear” (54, 55). However, whereas studies report contradictory results with regards to hyper or hypo-cortisolism following early stress, it appears that the most consistent finding is reduction in the system’s variability and flexible response to both daily states and momentary stressors (56). Overall, our findings highlight the complexity of HPA functioning in the face of acute or chronic stress and the need for an integrative multi-level understanding of the vicissitudes of maternal psychopathology, associated stress, mother-child relationships, pituitary–adrenal-axis, and medulla interplay when attempting to tease apart the bio-behavioral mechanisms underlying some of the well-known devastating effects of maternal depression on children.

Limitations include the decision to exclude mothers with comorbid anxiety disorder. This may limit the generalizability of our results, since anxiety is highly comorbid with depression. Thus, future studies may be needed to look at a group of mothers suffering from depression with comorbid anxiety. Furthermore, although salivary cortisol and DHEA are well accredited methodologies these are peripheral measures and may not reflect actual central nervous system activity. In addition, paternal psychopathology was not assessed. Finally, our findings remain to be integrated with the vast network of variables that act within the central nervous system and our knowledge and ability to understand and work with the multiple arrays of factors involved is necessarily limited (27). The current findings shed further light on these complex systems and future research is needed to advance our efforts to help children of depressed mothers already in the first years of life through the construction of more specifically targeted early interventions. These results raise the intriguing possibility that in the future vicissitudes of DHEA regulation may be used as a biological marker for maternal sensitivity and/or the quality of the mother-child dyadic relationship. Such a marker could prove invaluable in the assessment of techniques to improve the maternal sensitive caregiving.

## Data Availability Statement

The datasets generated for this study will not be made publicly available because: privacy of subjects (minors) and their mentally-ill mothers.

## Ethics Statement

The studies involving human participants were reviewed and approved by Bar Ilan University Ethics Committee. Written informed consent to participate in this study was provided by the participants’ legal guardian/next of kin.

## Author Contributions

RF designed the study and conducted the follow-up and wrote the paper. YA-L conducted the 6-year follow-up, analyzed the data, and wrote the paper. OZ-S conducted the hormonal analysis.

## Funding

The study was supported by the Israel Science Foundation (08/1308), NARSAD independent investigator award to RF, the Irving B. Harris Foundation, and the Simms Foundation.

## Conflict of Interest

The authors declare that the research was conducted in the absence of any commercial or financial relationships that could be construed as a potential conflict of interest.
